# Meeting public health needs in emergencies–World Health Organization guidelines

**DOI:** 10.1111/jebm.12314

**Published:** 2018-08-09

**Authors:** Susan L. Norris

**Affiliations:** ^1^ Health Metrics and Measurement World Health Organization Geneva Switzerland

**Keywords:** emergency health services, practice guideline, public health, quality of care, World Health Organization

## Abstract

The World Health Organization (WHO) is a leading source of trustworthy guidelines in public health, including in emergencies. In addition to standard guidelines produced in preparation for emergency response, WHO has processes and methods for issuing guidelines in the context of urgent public health need, including rapid advice guidelines (production time 2 to 3 months) and health emergency interim guidelines (days to weeks). There are numerous challenges to producing guidelines in response to an emergency in addition to the compressed timeline which necessitates truncating or modifying standard processes. There is frequently a lack of scientific data on the disease or situation at hand, especially early in the event timeline. Resources are limited, particularly the availability of WHO staff and external experts, and disease and emergency response experts may lack knowledge and experience in developing guidelines. Finally, the rapid production of new information and the resultant short shelf‐life of recommendations pose a significant challenge to keeping guidelines up to date. In order to better meet end‐users’ needs, WHO must anticipate areas of uncertainty in emergency response and proactively develop relevant guidelines, explore optimal ways of communicating gaps in knowledge in the field to guideline developers, and promote and participate in research on the sources of bias in guideline development within compressed timeframes.

## 1 INTRODUCTION

The World Health Organization (WHO) is the United Nation's (UN's) directing and coordinating authority on international health for the UN's 194 Member States.^1^ WHO's core functions include: providing leadership on matters critical to health; shaping the research agenda and stimulating knowledge generation, translation, and dissemination; setting norms and standards and promoting and monitoring their implementation; articulating ethical and evidence‐based policy options; providing technical support; and monitoring health indicators.^1^ Thus the evidence ecosystem including its translation into evidence‐informed, trustworthy, and impactful recommendations and guidelines is central to WHO's mandate and vision.

WHO is determined to have a “strong and effective presence, particularly in countries prone to public health emergencies.”^2^ However, hard‐learned lessons during the Ebola Virus disease outbreak in West Africa from 2014 to 2016 have forced WHO to re‐examine its roles, organizational structure and governance, relationships with the Member States, operational efficiency, and its ability to provide timely, responsive and high‐quality technical normative guidance.^3^, ^4^ In addition, WHO is facing increasing demands for timely and effective emergency response as the number of acute public health emergencies that require an operational response by WHO increases.^5^, ^6^

Since its inception in 1948, WHO has produced a vast array of technical normative guidance across a broad range of topics, including those related to emergency response. WHO guidelines are of several types (Table 1): standard guidelines follow full processes, are based on systematic reviews of the relevant evidence, and involve meetings of external experts at which recommendations are formulated based on explicit criteria and processes. Because standard guidelines take 6 months to 2 years or more to develop, WHO staff have instituted processes and methods for developing guidelines in compressed timelines in response to urgent Member State need.

**Table 1:** Types of guidelines at the World Health Organization

Type of guideline	Estimated development time	Indication	Key characteristics
Standard guideline	6 months to 2 years	Non‐emergency settings and timelines	Follow standard approaches to systematic reviews and guideline development.
Rapid advice guideline	2 to 3 months	Established and ongoing emergencies where technical guidance is needed within several months and the recommendations can be rapidly implemented	These guidelines are based on rapid, systematic reviews of the evidence on very focused topics. They usually involve an expert meeting, either virtual or in‐person. A short shelf‐life is anticipated so these guidelines are usually labeled “interim” with a commitment to updating.
Health emergency interim guideline (HEIG)	Several days to 3‐4 weeks	Urgent need for technical guidance where no existing guidance exists and the recommendations can be rapidly implemented	The very short timeline and the frequent paucity of structured scientific evidence necessitate ultra‐rapid identification of key questions, the use of existing evidence syntheses and indirect evidence, virtual meetings, and reliance on expert opinion (which is explicit and transparent). These guidelines have a short shelf‐life so are labeled “interim” with a commitment to updating.

Note that these types of guidelines and their characteristics are part of a spectrum and the information presented here represents typical situations. Timelines, methods, and characteristics will vary with each emergency in order to best meet end‐users’ needs.

Two basic processes and document types can be developed in response to urgent public health need: rapid advice guidelines and health emergency interim guidelines (HEIGs). Although categorizations and labels are useful, these information products represent a spectrum from standard to “ultrafast” guidelines, as the development of every document is tailored to Member States’ and end‐user needs, and to the evidence that is available.

Rapid advice guidelines can be completed within 2 to 3 months using abbreviated and accelerated methods. These types of guidelines have well‐development processes, procedures and standards as described in the *WHO handbook for guideline development* (2nd edition, 2014),^7^ including a planning proposal, rapid reviews of the evidence on benefits and harms, a convening of experts to formulate recommendations according to specific criteria, targeted peer review, and review and approval by the internal quality oversight body for WHO guidelines (the Guidelines Review Committee [GRC]).

HEIGs are information products focused on knowledge gaps identified during an emergency response for which no relevant, up‐to‐date guidelines exist and the guidance is needed within a few days to several weeks. The GRC Secretariat developed steps and a toolkit for developing such guidelines, including linkages to WHO's Incident Management System to ensure appropriate prioritization of guidelines being produced; tips for focusing on the key areas of uncertainty in the field; interlinked templates for planning, development, approval and production such that information and text are only entered once, with all steps leading directly to the final information product; input from technical experts, field staff and end‐users, all of whose declarations of interests have been collected and any conflicts appropriately managed; targeted peer review; and GRC review (although not an approval process) with 24‐hour turn‐around.

Despite the wide variations in development time, all WHO guidelines adhere to the principles of trustworthy information products: use of explicit and transparent processes and methods; the application of as rigorous an approach as possible to minimize bias in evidence collection, appraisal and synthesis, and in the resultant recommendations; a focus on end‐users’ needs; and the production of usable, impactful guidance.

## 2 CHALLENGES

The production of high‐quality guidelines in response to public health emergencies poses a number of challenges for WHO technical units. First, the short time periods available for rapid advice guidelines and most particularly for HEIGs impose difficult decisions regarding truncating, omitting, or accelerating steps in the standard guideline development process. Each decision can potentially jeopardize the trustworthiness and accuracy of the resultant guideline. The approaches used to minimize the risks include standardized techniques to minimize bias in evidence collection and synthesis, collection and management of declaration of interests, early and continuous consultation with experts to identify relevant data, peer review, and involvement of a methodologist who is not a content expert and who has no pre‐conceived ideas about the “right” recommendation. However, there is little information in the guideline methods literature on the most important sources of potential bias to guide decisions on which corners to cut.

Second, there may be a paucity of structured scientific data on emerging diseases or novel situations, especially early in the event timeline, hindering the formulation of evidence‐informed recommendations. Unlike many standard guidelines which are often engendered by a plethora of evidence or significant new data, guidelines in emergencies are often solely driven by uncertainty and needs in the field. This situation makes it all the more important to adhere to structured, transparent and explicit processes for evidence identification and appraisal, and for the formulation of recommendations. On the other hand, the lack of research evidence simplifies the evidence collection and appraisal phase, and the expert group can move quickly to decision‐making, taking issues such as feasibility, acceptability, equity, and resource considerations into account in a more *ad hoc*, expert opinion‐based (but transparent) manner.

Third, resource limitations, particularly the availability of WHO staff and external experts is a critical issue. As we found in the Ebola outbreak of 2014‐2016, content experts, programme managers and field workers as well as WHO staff were focused on operational issues, emergency response management, and care delivery. Spending time contributing to a guideline was understandably of lower priority for these individuals.

Fourth, emergency response managers and staff may not be versed in standard approaches to evidence‐based decision‐making and guideline development. As experts in their field, they may not understand or appreciate the value of additional processes and procedures required to develop evidence‐informed, trustworthy and impactful guidelines.

Fifth, many of the information products required and issued in emergency response do not lend themselves well to standard guideline processes, even to processes adapted to compressed timelines. Each document may contain hundreds of very specific statements on clinical care (e.g., airway and fluid management, or surgical techniques) which are best considered good practice statements or operational guidance, which may not benefit from reviews of the evidence or elaborate development processes.

Finally, guidelines developed rapidly in response to emergencies need to be reassessed regularly for continued validity and for the addition of any new information. Thus, these guidelines are labeled as “interim” at WHO and a “review‐by” date is required in the guideline publication. This short shelf‐life poses challenges as resources may continue to be severely constrained if the outbreak or other public health emergency continues or if there are competing concurrent emergencies. Data may not have been collected in a rigorous manner and trials may not have been implemented, thus expert opinion and programmatic experiences may be the only basis for an update. In addition, needs change over the course of emergencies: for example, in the Ebola outbreak in West Africa in 2014‐2016, the health needs of survivors emerged during the course of the outbreak, necessitating new guidance, which had to be prioritized along with updates of existing guidance.

## 3 THE FUTURE

As WHO staff, we have learned much from recent emergencies about how to prioritize, manage, develop, and issue guidelines in this challenging context, and have made tremendous progress in this regard. Nonetheless, both WHO and the international guideline community have significant work to do to improve guidelines produced in response to emergencies.

First, most acute health needs requiring technical guidance in emergency response can be anticipated and guidelines planned and developed prior to an event as part of risk governance, resilience, and preparedness priorities. In this ideal scenario, technical units use established processes and methods for standard guidelines to produce trustworthy, high‐quality documents. WHO is working to focus its guideline efforts more on this preparation phase, although this is challenging given our resource constraints.

Second, one of the most challenging steps in guideline development, and arguably the most important, is getting the questions right. Whether for standard guidelines, rapid advice guidelines or HEIGs, guidelines processes and methods are designed to address a key area of *uncertainty*. This critical step must be carefully considered, with input from the affected Member States, and from managers and field workers who are on the ground dealing with the event. Care must be taken to craft the questions such that they not only reflect the uncertainty and need, but are answerable (albeit even with expert opinion).

Third, further research is needed on the sources of bias in guideline development within compressed timeframes, in order to work toward the optimal balance between rigor (and development time) and production of a valid, impactful guideline. Finally, WHO staff responsible for guidelines in emergencies need to be trained in the principles and methods for evidence‐informed decision‐making and need to recognize the value of these approaches even in the most challenging of response settings. Equally importantly, guideline methodologists need to respect the constraints faced by real‐world managers and frontline responders, and develop and implement processes and methods that add value, while not obstructing the urgent need for guidance in pursuit of the perfect guideline according to standards set for nonemergency situations.

WHO, as a trusted source of technical normative guidance in healthcare, produces a large number of hugely impactful information products in the context of public health emergencies. We have made tremendous progress towards optimizing efficient production, transparency, validity, and impact on health outcomes. However, challenges remain which the Organization and the international guideline development community need to continue to work diligently to address.
